# Role of Subclinical Gut Inflammation in the Pathogenesis of Spondyloarthritis

**DOI:** 10.3389/fmed.2018.00063

**Published:** 2018-05-01

**Authors:** Aroldo Rizzo, Giuliana Guggino, Angelo Ferrante, Francesco Ciccia

**Affiliations:** ^1^Dipartimento Biomedico di Medicina Interna e Specialistica, Università degli studi di Palermo, Palermo, Italy; ^2^Unità Operativa di Anatomia Patologica, Azienda Ospedaliera Ospedali Riuniti Villa Sofia Cervello, Palermo, Italy

**Keywords:** spondylitis, gut microbiome, inflammation mediators, innate immune response, enthesitis-related arthritis

## Abstract

Subclinical gut inflammation occurring in patients affected by spondyloarthritis (SpA) is correlated with the severity of spine inflammation. Several evidences indicate that dysbiosis occurs in SpA, and that may modulate intestinal permeability and intestinal immune responses. The presence of intestinal dysbiosis is accompanied in SpA patients with the presence of zonulin-dependent alterations of gut-epithelial and gut-vascular barriers. The leakage of epithelial and endothelial surface layers is followed by the translocation of bacterial products, such as lipopolysaccharide and intestinal fatty acid binding protein, in the systemic circulation. These bacterial products may downregulate the expression of CD14 on circulating monocytes leading to an “anergic” phenotype. In the gut, IL-23 may induce the expansion of innate immune cells such as mucosal-associated invariant T cells, γδ T cells, and innate lymphoid cells of group 3 that through the interaction with MAdCAM1 may recirculate form the gut to the sites of SpA active inflammation. On the basis of these findings, gut inflammation observed in SpA patient seems to be not only an epiphenomenon of the on going systemic inflammatory process but may also represent the base camp in which inflammatory cells are activated and from whom they shuttle.

## Introduction

**Figure 1:**
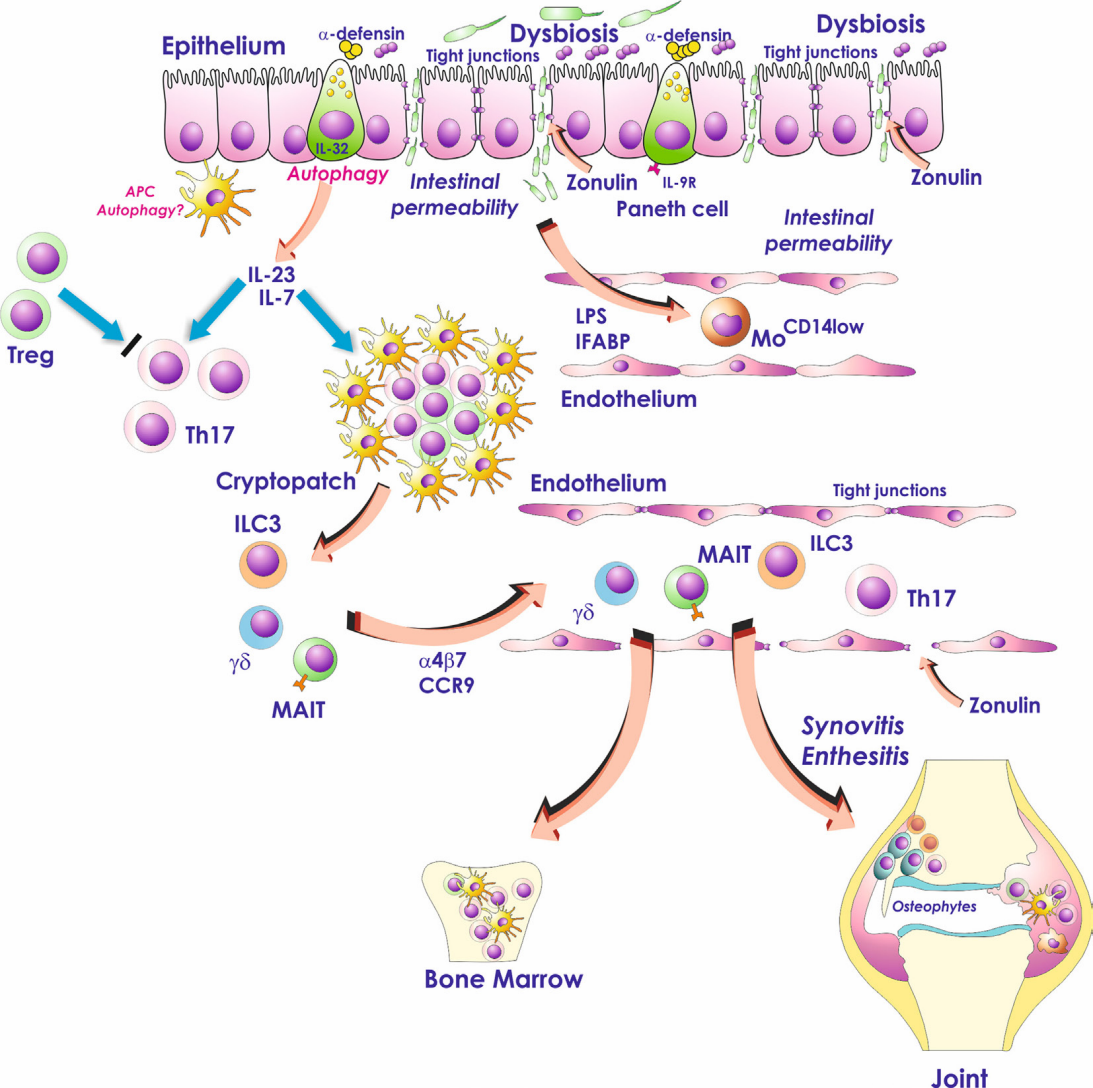
Role of the gut inflammation in the pathogenesis of ankylosing spondylitis. Dysbiosis, occurring in the gut of AS patients, activates Paneth cells to produce antimicrobial peptides and possibly modulates the production of IL-23. Dysbiosis is also associated with zonulin-dependent alteration of both gut-epithelial barrier and gut-vascular barrier. The presence of altered epithelial and endothelial permeability is followed by the translocation in the systemic circulation of bacterial products such as lipopolysaccharide (LPS) and intestinal fatty acid binding protein (iFABP). These bacterial products may downregulate the expression of CD14 on circulating monocytes leading to an “anergic” phenotype. In the gut, IL-23 may induce the expansion of innate immune cells such as mucosal-associated invariant T (MAIT) cells, gd T cells, and innate lymphoid cells of group 3 (ILC3) that through the interaction with MAdCAM1 may recirculate form the gut to the sites of AS active inflammation.

Subclinical intestinal inflammation occurs in a significant number of patients affected by spondyloarthritis (SpA) and is correlated with the severity of spine inflammation (1, 2). Different studies indicate the occurrence of an altered microbiome (so-called dysbiosis) in the intestine of patients with SpA, associated with profound alterations of both gut-vascular barrier (GVB) and gut-epithelial barrier (Figure 1). The interaction between intestinal bacteria and the gastrointestinal immune system might drive in SpA the aberrant activation of innate immune cells that seem to be able to recirculate from the gut to the sites of SpA extraintestinal inflammation (Figure 1). On the basis of these findings, gut inflammation observed in SpA patient seems to be not only an epiphenomenon of the on going systemic inflammatory process but may also represent the base camp in which inflammatory cells are activated and from whom they shuttle (Figure 1). In this review, we will talk only about subclinical gut inflammation and SpA. The problem of the occurrence of clinically evident gut inflammation in SpA patients is more complex. Theoretically, we should consider such a patient as concomitantly affected by inflammatory bowel disease (IBD) and spondylitis. Our preliminary studies demonstrate that this kind of intestinal inflammation is immunologically more similar to Crohn’s disease (CD) than subclinical gut inflammation and requires more studies to be defined.

## The Histopathology of Subclinical Intestinal Inflammation in SpA

Two main histologic varieties of subclinical intestinal inflammation have been described in the gut of SpA patients, the acute and the chronic type of inflammation (1). Acute inflammation is histologically similar to bacterial enterocolitis, characterized by the lamina propria infiltration with polymorphonuclear cells (1). The chronic type of inflammation, resembling CD is characterized by crypts distortion with villous atrophy, flattening and fusion of villi, increased infiltration of lamina propria mononuclear cells (LPMCs) and lamina propria lymphoid follicles (1). More recently, our group has demonstrated the occurrence of specific histologic changes in SpA gut independently by the degree of intestinal inflammation. These alterations include goblet cells hyperplasia and increased mucins production and activation of Paneth cells (3). The natural history of subclinical gut inflammation in SpA in not clear even though it has been demonstrated that up to 10% of those patients displaying chronic gut inflammation will develop over the time a clinically overt CD (4). Further studies are required to clarify whether acute and chronic forms are a continuous histologic spectrum of the same disease and/or a transition from one form to another may occur.

## Dysbiosis in SpA Gut

The microorganisms that colonize the human body are collectively termed the microbiome (5). The compositional and functional alteration in the microbiome, driven by a set of environmental- and host-related factors, is defined as dysbiosis (5). Several evidences indicate that dysbiosis occurs in SpA. HLA-B27 is one of the most important genetic risk factor for patients with SpA and the role of HLA-B27 in modulating the intestinal microbiome has been recently studied by the Rosenbaum’s group (6). The authors demonstrated that that Lewis rats transgenic for HLA-B27 and human β2-microglobulin had significant differences in the cecal microbiota compared with wild-type Lewis rats (6). Ciccia et al. (3) demonstrated that both adherent and invading Gram^+^ and Gram^−^ bacteria are observable in the inflamed ileum of SpA patients and are always associated with histological alterations characterized by the epithelial cell detachment, forming subepithelial vacuoles, and the presence of lamina propria edema with red blood cells extravasation.

Costello et al. (7) performed bacterial community profiling of terminal ileum biopsies of SpA patients. Ileal microbiome of patients with SpA differed was significantly different from healthy controls, with higher representation of Lachnospiraceae, Veillonellaceae, Porphyromonadaceae, and Bacteroidaceae and reduction of Prevotellaceae, Gemellaceae, Streptococcaceae, and Actinomycetaceae. A quantitative metagenomics study has been recently performed, based on shotgun sequencing of gut microbial DNA obtained from 211 Chinese patients (8). SpA patients showed a significant increased abundance of *Prevotella melaninogenica, Prevotella copri*, and *Prevotella* sp. C561 and decreased representation of *Bacteroides* spp. Interestingly, the *Bifidobacterium* genus, commonly used in probiotics, was observed to accumulate in the SpA patients. 16S ribosomal RNA gene sequencing has been recently also performed on fecal DNA isolated from stool samples in SpA by Breban et al. (9). The authors evidenced that a disease-specific dysbiosis was present in SpA. The most striking change was a twofold to threefold increased abundance of *Ruminococcus gnavus* in SpA, as compared with both RA and HCs that was significantly correlated with the disease activity only in patients with a history of IBD. It is noteworthy that among healthy controls, significant difference in microbiota composition was also detected between HLA-B27^+^ and HLA-B27^−^ siblings, indicating that the genetic background may influence the microbiota composition. Tito et al. (10) recently demonstrated that the type of intestinal inflammation, normal vs acute or chronic inflammation, is associated with the profile of mucosal microbiota in patients with SpA. In particular, in the inflamed biopsy tissues the bacterial community composition was completely different compared with non-inflamed biopsy tissues. The authors also found that the abundance of the *Dialister* genus was correlated with the Ankylosing Spondylitis Disease Activity score.

## Role of Dysbiosis in Modifying Gut Permeability in As

In healthy subjects, the intestinal microbiota cannot access the peripheral tissues and/or the systemic circulation avoiding the induction of systemic immune responses. Such compartmentalization is guaranteed by the presence of an epithelial gut barrier (11, 12) and of a GVB (13) controlling the translocation of antigens into the blood stream at the same time prohibiting the translocation of bacteria and/or bacterial products. The gut-epithelial barrier is constituted by a complex system of protein–protein networks linking the contiguous cells and closing the intercellular space (12). The network of protein that connects epithelial cells includes desmosomes, adherens junctions, and tight junctions (12). Recently, Spadoni et al. show that enteroglial cells and pericytes are in close contact with intestinal vascular endothelial cells and are an integral part of a GVB highly resembling the blood–brain barrier (BBB) (13). Like the BBB, endothelial cells in the GVB develop tight junctions allowing the diffusion of molecules as large as 4 kD, eight times the maximal size observed for the BBB. Gut-epithelial barrier and GVB were recently studied in the gut of SpA patients by Ciccia et al. (3). In this study, the presence of adherent and invading bacteria was associated with a profound downregulation of the tight junction proteins claudin 4 and occludin. The downregulation of tight junction proteins was associated in SpA with the upregulation of zonulin, the protein that has been demonstrated to modulate the permeability of tight junctions between intestinal epithelial cells (14) (Figure 1). Zonulin expression in SpA was inversely correlated with claudin 1, claudin 4, occludin, and zonula occludens and modulated by intestinal bacteria as demonstrated by the evidence that isolated ileal bacteria from patients with SpA induced a significant upregulation of zonulin on Caco-2 cells (3). The altered intestinal epithelial permeability was accompanied by the alteration of the GVB in SpA patients (Figure 1). VE cadherin, the most relevant endothelial adhesion molecule, and JAM-A, a vascular tight junctions protein, were significantly downregulated in the gut of patients with SpA together with the upregulation of PV1, a marker of endothelial cells permeability, especially in those patients with chronic gut inflammation (3). Confocal microscopy analysis of SpA ileal samples showed the disappearance of the classic endothelial continuous staining of CD31, a marker of endothelial cells and of glial fibrillar acid protein a marker of glial cells, confirming the disorganization of GVB (3). Alterations of epithelial and endothelial layers permeability, was associated with increased serum levels of lipopolysaccharide (LPS), LPS-BP and intestinal fatty acid binding protein. The presence of high LPS concentration was associated in SpA with the significant downregulation of the CD14, the co-receptor for LPS, expression on circulating monocytes (3). The findings coming from this study do not allow clarifying whether or not the intestinal bacteria might induce these alterations. However, HLA-B27 rats display ileal inflammation characterized by the downregulation of occludin and the presence of adherent bacteria (3). Antibiotics treatment caused the normalization of occludin expression and the disappearance of adherent bacteria suggesting a role of intestinal microbiome in modulating the integrity of epithelial barrier (3).

## Dysbiosis may Modulate IL-23 Production in as Gut

The inflamed gut of SpA patients has been demonstrated to be the main site where the production of IL-23 takes place (15). The demonstration that in HLA-B27 transgenic rats the antibiotics treatment induces the normalization of IL-23 expression might suggest a role of dysbiosis in the modulation of IL-23 production (3). The regulation of IL-23 in SpA gut is, however, not completely understood and different pathways of innate immune system might be implicated in IL-23 modulation. Autophagy is one of these innate immune system pathways targeting intracellular bacteria, in the process termed xenophagy, for lysosomal degradation (16). Autophagy is significantly modulated in the gut of AS patients with a strong upregulation of autophagy related 16-like 1 (ATG16L1), immunity-related GTPase family (IRGM) and microtubule-associated proteins 1A/1B light chain 3A (17). These alterations were more profoundly observed in patients with more severe gut inflammation and significantly correlated with IL-23p19. In this study, the inhibition of both autophagy and chaperone mediated autophagy was required, in the presence of LPS, to reduce the numbers of IL-23 expressing cells and to increase the IL-23p19 mRNA levels in the LPMCs (17) (Figure 1). The intestinal activation of autophagy in SpA patients seems to be a tissue-specific phenomenon since that it is not present in the peripheral blood (18), the synovial tissues (18) and the spine (19) of SpA.

## IL-23-Dependent Innate Immune Responses in SpA

IL-23 overexpression has been demonstrated to immunologically characterize the subclinical inflamed gut of SpA patients (15). In SpA gut, IL-23 is mainly produced by infiltrating myeloid cells and by a subset of highly specialized epithelial cells, located at the base of crypts, the so-called Paneth cells (15). IL-23 production has been proved to constitutively occur in the terminal ileum of normal subjects (20), suggesting a homeostatic role of this cytokine in the modulation of gut immune responses. In the gut of SpA patients, differently from the gut of PsA (21) and Behcet’s disease (22) patients, IL-23 polarization seems to be not accompanied by the presence of a clear Th17 polarization (15), possibly as the results of the occurrence of a strong Treg response (23). IL-23, however, is able by itself to induce intestinal inflammation (24) and may also induce the expansion and activation of different subsets of innate immune cells such as innate lymphoid cells type 3 (25, 26), γδ T cells (27), and mucosal-associated invariant T cells (28) (Figure 1).

Mucosal-associated invariant T cells are a subset of T lymphocytes characterized by the expression of a semi-invariant T cell receptor alpha (TCRα) chain (29). The TCRα arises from the rearrangement of the variable (V) and joining (J) TCRα segments during VDJ recombination. MR1, an MHC class I-like protein, is required for the presentation of bacterial-derived vitamin B metabolites to MAIT cells modulating the release of several pro-inflammatory cytokines and the ability of killing bacterially infected cells. In mice, MAIT cells are activated by IL-23 and IL-1b in the absence of TCR stimulation (30). In humans, both IL-7 and IL-23 are able to induce MAIT cells polarization resulting in a different modulation of STAT3, HIF1alpha and RORc (31). IL-7 stimulation induces in fact a significant STAT3 and HIF1alpha upregulation with any relevant modulation of RORc. Conversely, IL-23 stimulation significantly induced RORc overexpression not affecting STAT3 and/or HIF1alpha expression (31). MAIT cells’ development and activation mainly occur in the gut and the presence of gut inflammation in a significant percentage of SpA patients may strengthen the existence of a gut–joint axis of inflammation in AS. Although no data are available regarding MAIT cells frequency and function in SpA gut, the frequency of MAIT cells is reduced in the blood of SpA patients (28). Furthermore, patients with SpA have an increased frequency IL-17A^+^ MAIT cells and IL-17^+^ MAIT cells are enriched in SpA synovial fluids (28). However, IL-17 production in SpA peripheral MAIT cells seems to be dependent by IL-7 but not IL-23 or antigen stimulation (28).

Gamma delta T cells are a subset of T cells in which the genetic composition of TCR is encoded by the gamma and delta gene loci, enriched in epithelial cell-rich compartments such as the digestive tract (32). Subsets of γδ T cells are differentiated on the basis of the inclusion of invariant TCR V-(D)-J segments showing tissue or context specificity. γδ T cells represent a fundamental link between adaptive and innate immune responses. They may in fact undergo to V-(D)-J segment rearrangement inducing adaptive, antigen-specific responses. However, they can also be directly activated *via* the recognition of pathogen-associated molecular patterns (PAMPs) or danger-associated molecular patterns, by the gamma delta TCR or non-TCR proteins. Like T helper cells, γδ T cells produce different effector pro-inflammatory cytokines in a subtype and context-specific way. Recently, cocultures of naive Vγ9Vδ2 T cells with phosphor antigens and pro-inflammatory cytokines, such as IL-1-β, TGF-β, IL-6, and IL-23, have been demonstrated to potently induce the polarization toward IL-17 production by expressing the transcription factor RORγt (33). In a murine model of SpA, activated Vγ6^+^CD27^−^ γ/δ T cells were expanded in non-inflamed entheseal tissues representing the large majority of RORγt^+^IL-23R^+^ entheseal-resident lymphocytes (34). In the presence of inflammations, the numbers of γ/δ T cells increased in the Achilles tendon enthesis, aortic root, and near to the ciliary body (34). In SpA patients, an increased frequency of circulating IL-17^+^IL-23R^+^ γ/δ T cells has been also recently demonstrated (27). In this study, the frequency of circulating IL-23R^+^ T cells was twofold higher in SpA patients, being γ/δ T cells the most relevant IL-23R^+^ population observed in SpA patients. The increased expression of IL-23R on γ/δ T cells was associated with an augmented IL-17 secretion. Interestingly, no observable IL-17 production was found among IL-23R-negative γ/δ T cells in SpA. Furthermore, stimulation of γ/δ T cells from SpA patients with IL-23 and/or anti-CD3/CD28 strongly induced a skewed polarization toward IL-17 production.

ILCs are specialized effector cells characterized by the absence of recombined antigen-specific receptors, the absence of specific markers associated with other immune cell lineages and a lymphoid morphology (35). ILCs have been involved in the innate immunity and inflammation regulation through the secretion of specific cytokines and chemokines. Like effector T cells, ILCs are divided into three main groups [ILC1, ILC2, and innate lymphoid cells of group 3 (ILC3)] based on the expression of specific transcription factors and cytokine production. ILC1, similar to Th1 cells, express the transcription factor Tbet and produce high amount of IFN-γ. Like Th2 cells, ILC2 produce IL-5 and IL-13 and are involved in allergy and mucosal homeostatic responses. ILC3, expressing the retinoic acid-ROR-γ and/or Tbet, are an important source of IL-22 and IL-17. ILC3 are IL-23-responsive immune cells and play a pro-inflammatory role also mediating protective responses against extracellular bacterial infections and providing help to marginal zone B cells through the production of BAFF. ILC3 depend on IL-7 for their differentiation and are essentially mucosal-restricted cells (36). In the gut of AS patients, ILC3 expressing the natural cytotoxicity receptor NKp44 are expanded and are characterized by the intense production of IL-17 and IL-22. NKp44^+^ ILC3 (defined as Lyn^−^IL-23R^+^NKp44^+^Tbet^+^RORc^−^ cells) are expanded in the gut of patients with SpA and were significantly correlated with the disease activity (26). ILC3 are developmentally related to lymphoid tissue inducer (LTi) cells (37). It has been demonstrated that aggregates of LTi cells, the so-called cryptopatches, found in the intestinal lamina propria, are the structure responsible for the maturation of ILC3 in response to the gut microbiota (38). Interestingly, the presence of LTi aggregates, highly resembling for cryptopatches, have been recently found in the small intestine of patients with SpA in close proximity to intestinal crypts and Paneth cells (26). In the gut of SpA, Paneth cells have been demonstrated to produce IL-7 and the coculture of LTi with isolated epithelial cells strongly induces the differentiation of ILC3 and the expression of IL-17 and IL-22 (26). These results may suggest a fundamental role of intestinal dysbiosis in activating intestinal innate immune responses in the gut of SpA patients, finally resulting in the differentiation and activation of ILC3.

Despite their mucosal restriction, ILC3 were also expanded in the peripheral blood, synovial fluids and the bone marrow (BM) tissues of SpA patients (26). Interestingly, the majority of these cells express the intestinal homing integrin α4β7. According to the α4β7 increased expression, the α4β7 ligand MAdCAM1, was highly represented in the context of SpA intestinal and BM high endothelial venules (26). More recently, ILC3 have been specifically studied in human enthesis (39). The proportion of ILC3 in human entheseal tissue was higher than that in peripheral blood and entheseal tissue had a higher proportion of NKp44^+^ ILC3s. Altogether these findings may support the idea that a gut–spine axis occur in SpA patients suggesting a role for MAdCAM1 in attracting ILC3 in the site of active AS inflammation and the recirculation of ILC3 between the gut and the inflamed spine. This concept seems to be supported by the findings that a4b7 block by natalizumab may be effective in ameliorating signs and symptoms in SpA patients (40).

It is interesting to note that blocking of IL-23 seems to be less effective in patients with SpA and pure axial disease compared with patients with peripheral disease (41). Since the evidences that blocking IL-17 significantly modifies the progression of axial disease in SpA patients we can assume that IL-22 and IL-17 are the key cytokines involved in the pathogenesis of AS.

## Conclusion

Spondyloarthritis subclinical gut inflammation may be considered the occult engine of the disease. In the gut, the complex interactions between the microbiome and the host immune system may lead to the alteration of intestinal barriers and to the aberrant activation of innate immune cells. Recirculating innate immune cells from the gut to the extraintestinal sites of inflammation may be responsible for the induction of chronic inflammatory responses in patients with SpA.

## Author Contributions

AR, FC, GG, and AF contributed equally in writing the review. AF prepared the figure.

## Conflict of Interest Statement

The authors declare that the research was conducted in the absence of any commercial or financial relationships that could be construed as a potential conflict of interest.
